# “Heart Sign” with Double Aortic Arch

**DOI:** 10.3390/diagnostics16142188

**Published:** 2026-07-14

**Authors:** Michael Scott Binder, David Majdalany

**Affiliations:** Mayo Clinic, Rochester, NY 55901, USA

**Keywords:** adult congenital, vascular ring, echocardiography

## Abstract

**Double** aortic arches are rare congenital malformations resulting from persistence of the right and left aortic arches, as opposed to normal regression of the left aortic arch. These vascular malformations can cause vascular rings since they encompass the trachea and esophagus, and lead to swallowing difficulties or respiratory difficulties from extrinsic compression. **Typically**, these are diagnosed in childhood but rarely may present in adults. In the presence of symptoms, which require formal gastrointestinal and pulmonary evaluation, surgical resection of the nondominant arch is typically recommended to release the vascular ring. Monitoring of the carotid and subclavian vasculature is performed intraoperatively to ensure distal perfusion is not affected following distal ligation of the arch.

**Figure 1:**
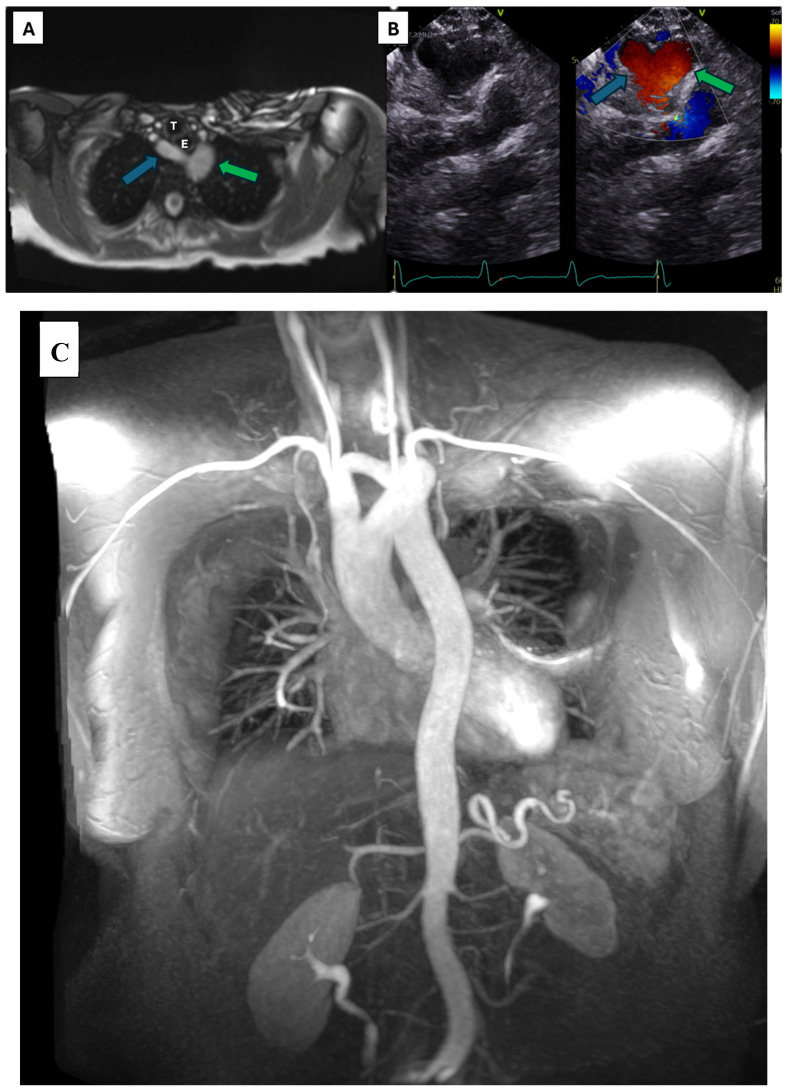
A 59-year-old female with past medical history of atrial fibrillation, sleep apnea and obesity had a cardiac MRI (**A**) which showed an incidental finding of a double aortic arch. There was a larger left arch (green arrow, 2 cm) and smaller right arch (blue arrow, 1.2 cm) which formed a complete vascular ring (trachea, T, and esophagus, E, on panel (**A**)). On evaluation, she had aversion to large medications for the past 30–40 years but no overt dysphagia. Her transthoracic echo (**B**) image taken at the suprasternal notch with probe marker at 3-o-clock illustrated a classic “heart sign” at the convergence of both aortic arches into the descending aorta. A 3-dimensional reconstruction from her MRA is also included (**C**). Complications are typically related to mechanical compression of the trachea and/or esophagus by the complete vascular ring and can increase in adulthood as the size of the aorta expands [1]. Typical next steps in testing include both pulmonary and barium swallow to assess dynamic tracheal or esophageal compression from the ring which may contribute to symptoms [1]. The patient underwent a barium swallow which demonstrated significant dysphagia to solid food. Treatment generally consists of surgical division of the nondominant arch by thoracotomy on that side, while ensuring that perfusion remains intact to both carotid and subclavian vessels [1,2]. Long-term outcomes are generally very favorable, with very low perioperative mortality and a complication rate of around 10% [3]. Persistent symptoms vary depending on the study but range around 33% [3]. These anomalies are typically diagnosed in utero on fetal ultrasound in the three-vessel view or as a child when symptoms develop, but very rarely can present de novo as an adult due to aortic expansion [4].

## Data Availability

All data is available upon request to the authors from certified researchers.
